# A Novel Method for Optimum Global Positioning System Satellite Selection Based on a Modified Genetic Algorithm

**DOI:** 10.1371/journal.pone.0150005

**Published:** 2016-03-04

**Authors:** Jiancai Song, Guixiang Xue, Yanan Kang

**Affiliations:** 1 School of Electronic Information Engineering, TianJin University, TianJin, China; 2 School of Computer Science and Technology, Tianjin University, TianJin, China; 3 School of Computer Science and Engineering, Shijiazhuang Tiedao University, HeBei, China; Beijing University of Technology, CHINA

## Abstract

In this paper, a novel method for selecting a navigation satellite subset for a global positioning system (GPS) based on a genetic algorithm is presented. This approach is based on minimizing the factors in the geometric dilution of precision (GDOP) using a modified genetic algorithm (MGA) with an elite conservation strategy, adaptive selection, adaptive mutation, and a hybrid genetic algorithm that can select a subset of the satellites represented by specific numbers in the interval (4 ∼ *n*) while maintaining position accuracy. A comprehensive simulation demonstrates that the MGA-based satellite selection method effectively selects the correct number of optimal satellite subsets using receiver autonomous integrity monitoring (RAIM) or fault detection and exclusion (FDE). This method is more adaptable and flexible for GPS receivers, particularly for those used in handset equipment and mobile phones.

## Introduction

The geometric dilution of precision (GDOP) [1, 2] describes the accuracy of positioning from the geometry of the satellites visible to the receiver [3]. Errors in a GPS receiver’s determination of its position are typically the GDOP times the measurement error; in other words, the GDOP is an error amplification factor, which means that a smaller GDOP generally results in a more accurate position. Therefore, a smaller GDOP is better. It has been shown that the number of satellites cause the GDOP to move in the opposite direction, i.e., a higher number of satellites leads to a lower GDOP value, and a lower number of satellites generally produces a bad GDOP [4] and results in a corresponding reduction in the positioning error. The simplest method is to choose a combination of all of the satellites, all of which must be in view to achieve optimal position accuracy with a minimal GDOP [5]; however, some GPS receivers can receive and parse navigation signals from only a limited number of satellites in real time because of restrictions on hardware resources. Therefore, at least four satellites should be selected for three-dimensional positioning to balance hardware resources and positioning performance, however, a minimum of 5 satellites is required to evaluate the consistency and reliability for the GPS receiver autonomous integrity monitoring(RAIM), meanwhile, more than five pseudo-ranges are need to be used to identify the contaminating measurement in the procedure of fault detection and exclusion (FDE).

Calculating the GDOP typically involves multiple mathematical matrix operations, such as inversions and transformations, particularly for the optimal satellite selection method, which involves selecting the subset with the lowest GDOP using all of the satellites. However, the approach used for GDOP calculation creates a heavy burden for the receiver because the calculation’s complexity increases exponentially with the number of visible satellites [5].

Satellite selection is a classical combinatorial optimization problem, the solution methods of which have been the subject of intense study in recent years. One solution method includes geometric methods, such as the fast satellite selection approach [6–8], quasi-optimal satellite selection [9], and optimal selection [10, 11]. Miaoyan Zhang [8] proposed a novel algorithm based on selecting the subset of in-view satellites that has a closer distance to optimal solution from the aspect of geometry when there are more than four satellites for multiple constellations.

The other solution methods include statistical and machine learning approaches based on neural networks [5, 12–15], support vector machines [16] and fuzzy logic [17]. Simon [12] initially proposed the neural network-based approach as a way to predict the GDOP and complete the classification, and back-propagation neural networks and optimal interpolative nets have been employed to achieve the two objectives, respectively. Jwo [5] proposed several types of neural network mapping relationships for different classes of relationships between inputs and outputs and compared the performance of different network architectures. Mosavi [13, 14] studied the relationships of eigenvalues between the visibility matrix and its inverse matrix for GDOP. Azami [15] developed several improved neural network training algorithms, including the Levenberg Marquardt (LM), modified LM and resilient BP (RBP) methods. ChihHung [16] addressed the approximation of the GPS GDOP using support vector machines (SVMs).

In this paper, we develop a novel modified genetic algorithm to select the optimal subsets of satellites for GPS receivers. Because of the global optimization capability and fast convergence of the modified genetic algorithm, this approach effectively searches the entire solution spaces and determines the optimal satellite subset after evolving through a number of generations without the number limitation of required visible satellites, which improve the adaptability and flexibility of the algorithm under a unified framework.

## Materials

### The Geometric Dilution of Precision

The mathematical model behind GPS is based on pseudo-range measurements between the receiver and visible satellites [4]. Without loss of generality, supposing that (*x*_*u*_, *y*_*u*_, *z*_*u*_) represents the three-dimensional coordinates of the receiver’s position in the ECEF system, (*x*_*j*_, *y*_*j*_, *z*_*j*_) denotes the coordinates of the *j*th satellite’s position in the ECEF system, and *t*_*b*_ indicates the equivalent distance of the time difference between the receiver arrival time and the satellite signal sent time. The receiver acquires information from *n*(*n* > 4) satellites and determines the pseudo-range value.

$$\rho_{j}={\left({\left(x_{j}-x_{u}\right)}^{2}+{\left(y_{j}-y_{u}\right)}^{2}+{\left(z_{j}-z_{u}\right)}^{2}\right)}^{1/2}+ct_{b}\;\left(j=1,2,\cdots ,n\right)$$ (1)

It is shown that Eq (1) is a nonlinear function because of the square root mathematical operation. In general, this class of problem can be linearized using the mathematical principle of Taylor series and be written in a concise matrix form as

$$\begin{array}{c} \Delta \rho \approx G\Delta x=G\Delta x+\epsilon \end{array}$$ (2)

where the order of measurement matrix *G* is *n* × 4 (*n* ≥ 4), which can be represented as

$$\begin{array}{c} G=\left(\begin{array}{cccc} a_{x_{1}} & a_{y_{1}} & a_{z_{1}} & 1\\ a_{x_{2}} & a_{y_{2}} & a_{z_{2}} & 1\\ \cdots & \cdots & \cdots & \cdots \\ a_{x_{n}} & a_{y_{n}} & a_{z_{n}} & 1 \end{array}\right) \end{array}$$

To simplify this problem, we assume that the measurement error has a Gaussian distribution independently. The least squares solution for the super definite matrix is typically given by

$$\begin{array}{c} \Delta x={\left(G^{T}G\right)}^{-1}G^{T}\Delta \rho \end{array}$$ (3)

To illustrate the position accuracy, the covariance of Δ*x* is

$$\begin{array}{ll} cov\left(\Delta x\right) & =E\left[\left(\Delta x\right){\left(\Delta x\right)}^{T}\right]\\ & =E\left[{\left(G^{T}G\right)}^{-1}G^{T}\left(\Delta \rho \Delta \rho^{T}\right)G{\left(G^{T}G\right)}^{-1}\right]\\ & ={\left(G^{T}G\right)}^{-1}cov\left(\Delta \rho \right) \end{array}$$ (4)

It is reasonable to assume that all of the errors in the pseudo-range measurements are stationary random processes for short time intervals, which are independent and identically distributed with a variance of $\sigma_{uere}^{2}$. Let

$$\begin{array}{c} cov\left(\Delta x\right)=\left(\begin{array}{cccc} \sigma_{x_{u}}^{2} & \cdot & \cdot & \cdot \\ \cdot & \sigma_{y_{u}}^{2} & \cdot & \cdot \\ \cdot & \cdot & \sigma_{z_{u}}^{2} & \cdot \\ \cdot & \cdot & \cdot & \sigma_{ct_{b}}^{2} \end{array}\right) \end{array}$$ (5)

The GDOP, which represents the amplification of the equivalent ranging errors in the measurement into the receiver’s position solution, is

$$\begin{array}{c} GDOP=\sqrt{\sigma_{x_{u}}^{2}+\sigma_{y_{u}}^{2}+\sigma_{z_{u}}^{2}+\sigma_{ct_{b}}^{2}}/\sigma_{uere}^{2} \end{array}$$ (6)

According to linear algebra and matrix theory [25], when *λ*_*i*_ are the eigenvalues of an invertible matrix *A*, $\lambda_{i}^{-1}$ are assumed to be the eigenvalues of the inverse of the matrix *A*^−1^. The measurement matrix $H={\left(G^{T}G\right)}^{-1}$ is always revertible, and the eigenvalues $\lambda_{i}^{-1}$ of $H={\left(G^{T}G\right)}^{-1}$ can be found using the eigenvalues *λ*_*i*_ of (*G* *^T^* *G*). This leads to a significant reduction in the number of calculations required to find the matrix inverse; the eigenvalues *λ*_*i*_ of (*G* *^T^* *G*) can be quickly calculated using QR decomposition. Let

$$\begin{array}{cc} GDOP= & \sqrt{tr({\left(G^{T}G\right)}^{-1})}\\ = & \sqrt{tr((H)}\\ = & \sqrt{\lambda_{1}^{-1}+\lambda_{2}^{-1}+\lambda_{3}^{-1}+\lambda_{4}^{-1}} \end{array}$$ (7)

where *tr*(⋅) represents the trace function of the matrix and *c* is the constant for the speed of light.

The satellite selection process is to select a subset of satellites visible in the current view for the purpose of the best positioning accuracy, which corresponds to selecting the rows of the visibility matrix *G* that produce the minimum GDOP as follows:

$$\begin{array}{c} S=\left\{s_{i}\right\},1\leq i\leq k,k\in \left(4,5,6,7\right) \end{array}$$ (8)

where *s*_*i*_ is the identification number of the satellite currently in view such that

$$\min_{\forall \left(G_{i},G_{j},G_{l},G_{m}\right)\in G}\;\left(GDOP\right)=\sqrt{tr\left({\left(G_{k}^{T}G_{k}\right)}^{-1}\right)}=\sqrt{\lambda_{1}^{-1}+\lambda_{2}^{-1}+\cdots +\lambda_{k}^{-1}}$$ (9)

### Genetic Algorithm Preliminaries

A genetic algorithm (GA) [18–20] is a robust global optimization approach that operates according to the principles of evolution, such as inheritance, selection, crossover and mutation.

In a GA, chromosomes are used to encode a candidate solution, and the fitness function is the indication factor that represents the quality of the individuals. As the population evolves, its average fitness gradually increases based on the principle of the ‘survival of the fittest’; excellent genes that are more fit are usually selected stochastically. The selected individuals are operated through the process of the genetic operator, such as selection, crossover and mutation, until the algorithm reaches termination.

In previous studies, GA was primarily used for micro-strip antenna optimization [21, 22], resolving ambiguity [23] and multi-path mitigation [24] in satellite navigation. This paper proposes a novel modified genetic algorithm (MGA) for GPS satellite selection.

## Methods

Because individuals evolve from generation to generation, the optimal solution is found in a short period of time. Several strategies were used to improve the performance of the algorithm. First, the elitist strategy reserves the most fit gene of the previous generation to improve the average fitness of the next generation. Second, adaptive mechanisms are used to adjust the mutation and crossover rates to maintain each generation’s diversity. Finally, a hybrid method accelerates the convergence. Without loss of generality, we establish the following mathematical definitions:

*MaxGenNumber* represents the maximum number of evolution cycles.
*PopSize* represents the size of the population.
*Pop* represents the population.
*Ind*_*i*_ represents the *i*th chromosome.
*Fitness*(*Ind*_*i*_) represents the fitness of the *i*th chromosome.

### Representation

The integer code includes a clear description of the satellite selection problem with population diversity followed by binary formation.

It is assumed that *N* GPS satellites are visible in the current view, which are coded as (*k*_1_, *k*_2_, ⋯, *k*_*N*_). The objective is to select the four signals *Ind*_*i*_ = (*k*_*i*_, *k*_*j*_, *k*_*m*_, *k*_*n*_) that provide the minimum GDOP value and that satisfy the following constraint:

$$\left\{\begin{array}{c} k_{i}\leq k_{j}\leq k_{m}\leq k_{n}\\ 1\leq i\leq j\leq m\leq n\leq N \end{array}\right.$$ (10)

where the size of the solution space is $C_{N}^{4}$, *Ind*_*i*_ represents an individual, and *k*_*i*_ is a gene on the chromosome. Without loss of generality, we assume that the list of currently visible satellites is [2, 5, 7, 14, 15, 20, 21, 25] and possible solutions are [2, 5, 7, 14] or [2, 5, 7, 15]. A total of 5 or 6 satellites must be selected when considering RAIM and fail detection and exclusion (FDE), which means that the code’s dimension must increase to, for example, [2, 5, 7, 14, 15] or [2, 5, 7, 14, 15, 20].

### The Fitness Function

The fitness function is a special type of objective function that indicates the current number of individuals; a larger number is better in most cases. We minimize the GDOP as follows to obtain a more accurate position:

$$\begin{array}{c} Fit\left(t\right)=\frac{1}{\sqrt{tr({\left(G_{4}^{T}G_{4}\right)}^{-1})}} \end{array}$$ (11)

Note that according to Eq (11), the fitness function is monotonic and non-negative, and the number of calculations can be reduced by omitting the square root, which yields

$$\begin{array}{c} Fit^{\prime }\left(t\right)=\frac{1}{tr({\left(G_{4}^{T}G_{4}\right)}^{-1})} \end{array}$$ (12)

### Selection

During each generation, individuals are selected by the *Ps* probability to produce the offspring, in which the individuals with higher fitness have considerably higher probabilities of being selected.

We define the fitness selection operator as *T*_*s*_ : *S*^*PopSize*^ → *S*, where *S* represents the solution space and *PopSize* is the population size. Then,

$$\begin{array}{c} P\left\{T_{s}\left(Pop\right)={Ind}_{i}\right\}=P\{Ind_{i}\}=\frac{Fitness\left({Ind}_{i}\right)}{\sum_{k=1}^{PopSize}Fitness\left({Ind}_{k}\right)} \end{array}$$ (13)

where *Fitness*(*Ind*_*i*_) is an individual’s fitness and the probability distribution function *P*{*Ind*_*i*_} satisfies the following condition:

$$\left\{\begin{array}{c} P\left\{{Ind}_{i}\right\}\geq 0\\ \sum_{i=1}^{PopSize}P\left\{{Ind}_{i}\right\}=1 \end{array}\right.$$ (14)

The role of the selection operator is to select the individuals that reproduce stochastically; the roulette wheel selection method [18] based on a proportional-selection mechanism is the most widely used strategy in which a greater fitness implies a higher selection probability.

To reduce the time required to find the optimal solution, the elitist reservation strategy is used. Using a ranking based on the population’s fitness, the best chromosomes are reserved for the next generation. This process accelerates the algorithm’s convergence.

### Adaptive Crossover Operators

Crossover is a critical genetic operator that is also called recombination. In this process, a couple of solutions are selected to create offspring that inherit the characteristics from the “parents”. The process continues until a new population is created.

Without loss of generality, *T*_*c*_ : *S*^2^ → *S* is a stochastic map with cross probability *p*_*c*_ for the single-point crossover operator. ∀(*Ind*_1_, *Ind*_2_) ∈ *S*^2^, *Y* ∈ *S*, there is

$$P\left\{T_{c}\left(Ind_{1},Ind_{2}\right)=Y\right\}=\left\{\begin{array}{cc} \frac{kp_{c}}{Len} & Y\neq Ind_{1}\\ \left(1-p_{c}\right)+\frac{kp_{c}}{L} & Y=ind_{1} \end{array}\right.$$ (15)

where *Len* is the length of the chromosome.

In general, the cross probability *P*_*c*_ strongly influences the performance of the MGA.

**Fig 1:**
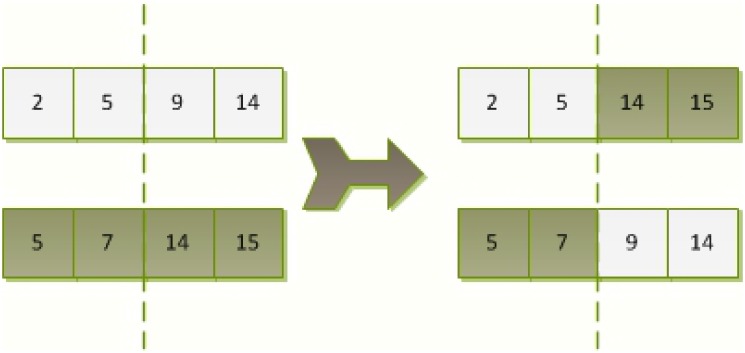
An example of the single-point crossover for MGA-based satellite selection.

During the initial stage of the MGA’s evolution, if the best chromosome is considerably more fit than the other individuals, the optimum chromosome has a substantially higher probability of being selected, which causes the algorithm to converge to a local minimum. Therefore, a gene with a high fitness should be restricted from over-reproducing to maintain the gene diversity at the initial stage of population evolution. In turn, the average fitness of all individuals is approximately equal to the maximum value of the population. Consequently, the possibility of selecting an individual with an average fitness is equal to that of selecting the chromosome with the highest fitness, which eliminates competition between excellent individuals. Then, the selection operation becomes random, which results in the worst performance when searching for the best chromosome during the post-evolutionary stage. Fig 1 is a demonstration of single-point crossover operation for MGA-based satellite selection.

The following novel adaptive strategy for generating the cross probability for different evolutionary stages that controls the gene diversity to a certain, but not large, degree is proposed:

$$P_{c}=\left\{\begin{array}{cc} P_{cmax}-\frac{\left(p_{cmax}-p_{cmin}\right)\left(Fitness^{\prime }-Fitness_{avg}\right)}{Fitness_{\max}-Fitness_{avg}} & Fitness^{\prime }\geq Fitness_{avg}\\ P_{cmax} & Fitness^{\prime }\leq Fitness_{avg} \end{array}\right.$$ (16)

where

*Fitness*_*max*_ and *Fitness*_*avg*_ denote the maximum and minimum fitness of the population, respectively. *Fitness*′ is the larger one between the pair of parents. *p*_*cmax*_ denotes the maximum probability of crossover. *p*_*cmin*_ denotes the minimum probability of crossover.

### Adaptive Mutation Operators

**Fig 2:**
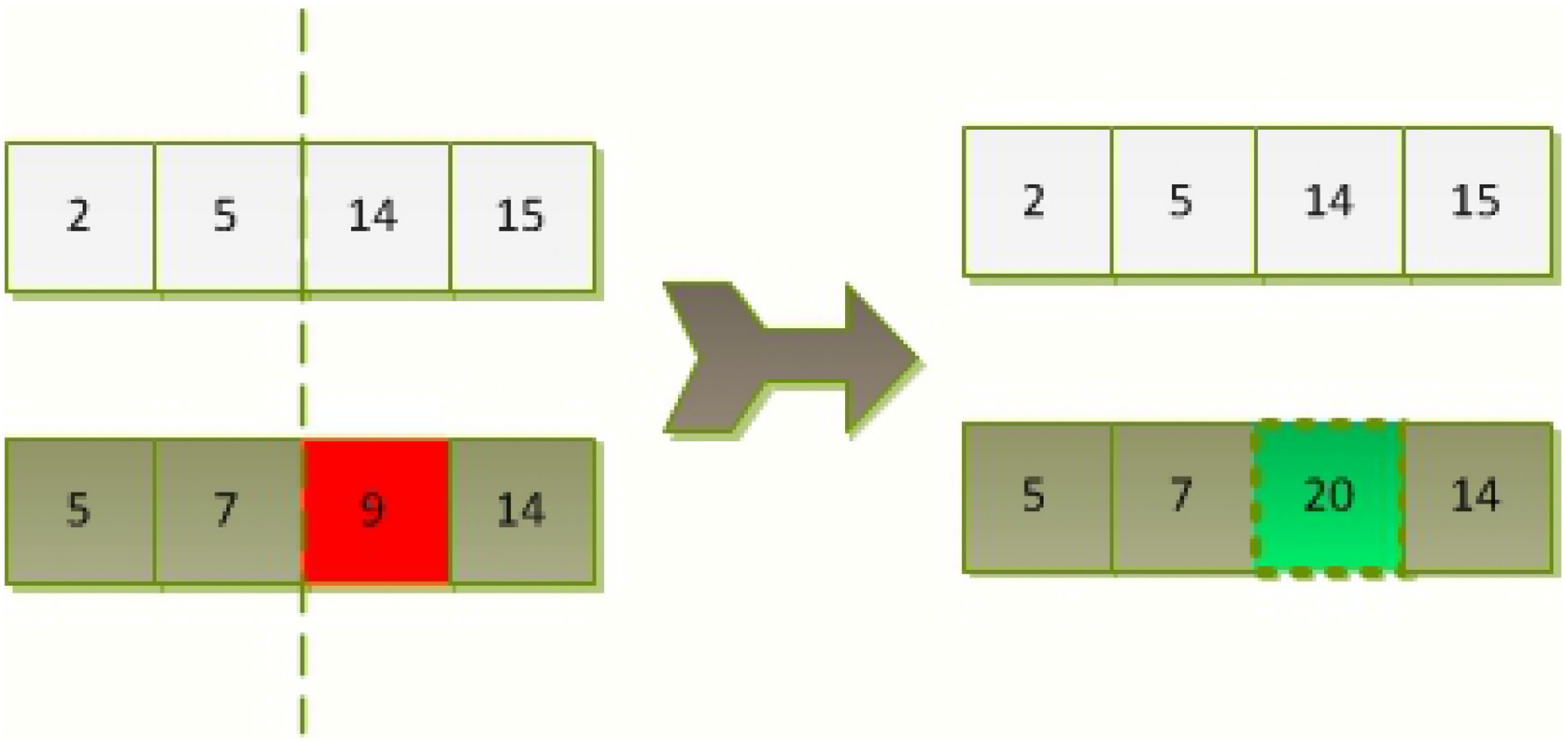
An example of the single-point mutation for MGA-based satellite selection.

The mutation operator brings a new gene into the population, keeping the population’s diversity from converging prematurely. Fig 2 is an example of single-point mutation operation for MGA-based satellite selection.

The mutation operator is used to alter an individual gene for the purpose of population diversity; however, the MGA degenerates into a random search when the mutation probability *P*_*m*_ is high. In contrast, the probability of producing certain useful genes is zero when *P*_*m*_ is low. To improve the algorithm’s ability to find a global optimum and to avoid converging prematurely or becoming stuck at a local minimum, the adaptive mutation is

$$P_{m}=\left\{\begin{array}{cc} P_{mmax}-\frac{\left(p_{mmax}-p_{mmin}\right)\left(Fitness^{\prime }-Fitness_{avg}\right)}{Fitness_{\max}-Fitness_{avg}} & Fitness^{\prime }\geq Fitness_{avg}\\ P_{mmax} & Fitness^{\prime }\leq Fitness_{avg} \end{array}\right.$$ (17)

where

*Fitness*_*max*_ and *Fitness*_*avg*_ denote the maximum and minimum fitness of the population, respectively. *Fitness*′ is the larger one between the pair of parents. *p*_*mmax*_ is the maximum probability mutation. *p*_*mmin*_ is the minimum probability mutation.

### The Hybrid Genetic Algorithm

To utilize the benefits of the two classes of approaches, a hybrid genetic algorithm that combines the advantages of an MGA and fast selection is proposed. To allow the algorithm to quickly find the best solution, individuals may be initially created in the areas with the higher probability for optimal solutions. It is easy to obtain an equivalent formulation of Eq (2) when the receiver position offset is described in the ECEF coordinate system.

$$\tilde{G}\;\left[\begin{array}{c} △e\\ △n\\ △u\\ △\delta t_{u} \end{array}\right]=b$$ (18)

where [△*e* △*n* △*u* △*δt*_*u*_]^*T*^ is the state variable to be determined; then, the geometry matrix $\tilde{G}$ becomes

$$\tilde{G}=\left[\begin{array}{cccc} -cos\theta^{\left(1\right)}sin\alpha^{\left(1\right)} & -cos\theta^{\left(1\right)}cos\alpha^{\left(1\right)} & -sin\theta^{\left(1\right)} & 1\\ -cos\theta^{\left(2\right)}sin\alpha^{\left(2\right)} & -cos\theta^{\left(2\right)}cos\alpha^{\left(2\right)} & -sin\theta^{\left(2\right)} & 1\\ \vdots & \vdots & \vdots & \vdots \\ -cos\theta^{\left(N\right)}sin\alpha^{\left(N\right)} & -cos\theta^{\left(N\right)}cos\alpha^{\left(N\right)} & -sin\theta^{\left(N\right)} & 1 \end{array}\right]$$ (19)

where *α*^(*n*)^ and *θ*^(*n*)^ are the elevation and azimuth angles of the *n*th satellite, respectively. The main idea behind this step is to randomly generate all of the chromosome subsets with geometries similar to that of the optimal subset of the population by grouping all of the satellites and randomly selecting one satellite from each group to form a chromosome subset according to their azimuths and elevations.

### Termination

This evolutionary process for MGA satellite selection arrives at an endpoint when one of the following conditions has been reached:

1. A solution is found that satisfies the minimum criterion, *GDOP* < 3.
2. The number of generations reaches 20.

## Results

To demonstrate the performance of the proposed MGA satellite selection algorithm, the OEMStar, which is one of NovAtel’s OEM global navigation satellite system receiver platforms, was used to collect broadcast ephemeris data and to calculate all of the satellites’ positions in the ECEF coordinate system every second for 12 hours. The OEMStar receiver was placed at the author’s institute, which has ECEF coordinates of [−2258692.95, 4405376.94, 4007987.94]. At the beginning of the evolutionary process, four different satellite signals were randomly selected to compute the GDOP from the geometry matrix G, using Eq (6). The following experiments were conducted using Matlab 2010 on a personal computer with an Intel Core(TM)2 Duo CPU and 2 GB of memory.

### Simulation Parameters

The primary parameters of the proposed MGA satellite selection algorithm include the population size, the crossover probability, the mutation probability and the number of evolution iterations. These parameters influence the convergence speed and accuracy of the algorithm. A high crossover probability accelerates the creation of individuals and increases the possibility of an excellent gene being destroyed, which negatively affects the process of evolution. In contrast, when the crossover probability is low, the speed at which new individuals are created decreases, and the search process stagnates. With regard to the mutation probability, a high probability results in the MGA being closer to a random search algorithm, and a low probability reduces the likelihood of producing new individuals and of premature convergence. Table 1 lists the values of all of the parameters used in the simulations.

**Table 1:** A summary of the parameter values used in this paper.

Notation	Meaning	Value
*PopSize*	Population size	60
*P*_*s*_	Selection probability	0.6
*GenNumber*	Maximum number of generations	20
*Ind*_*i*_	Chromosome: one solution to satellite selection problem	
*Len*	Length of chromosome	
*Fitness*(*Ind*_*i*_)	Fitness of chromosome	
*P*_*cmax*_	Maximum crossover probability	0.9
*P*_*cmin*_	Minimum crossover probability	0.6
*P*_*mmax*_	Maximum mutation probability	0.1
*P*_*mmin*_	Minimum mutation probability	0.005

## Discussion

**Fig 3:**
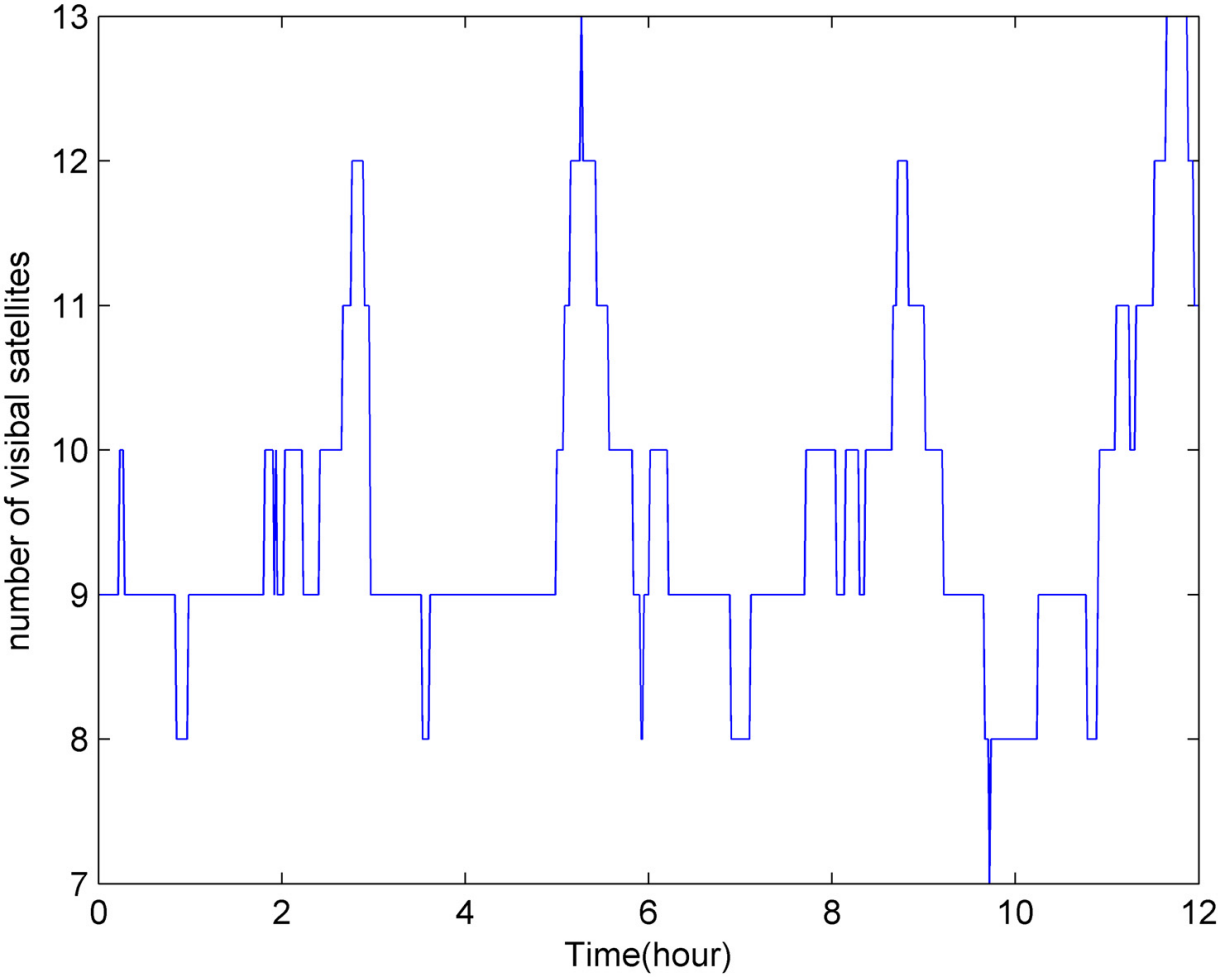
The number of GPS satellites visible from TianJin over 12 hours.

**Fig 4:**
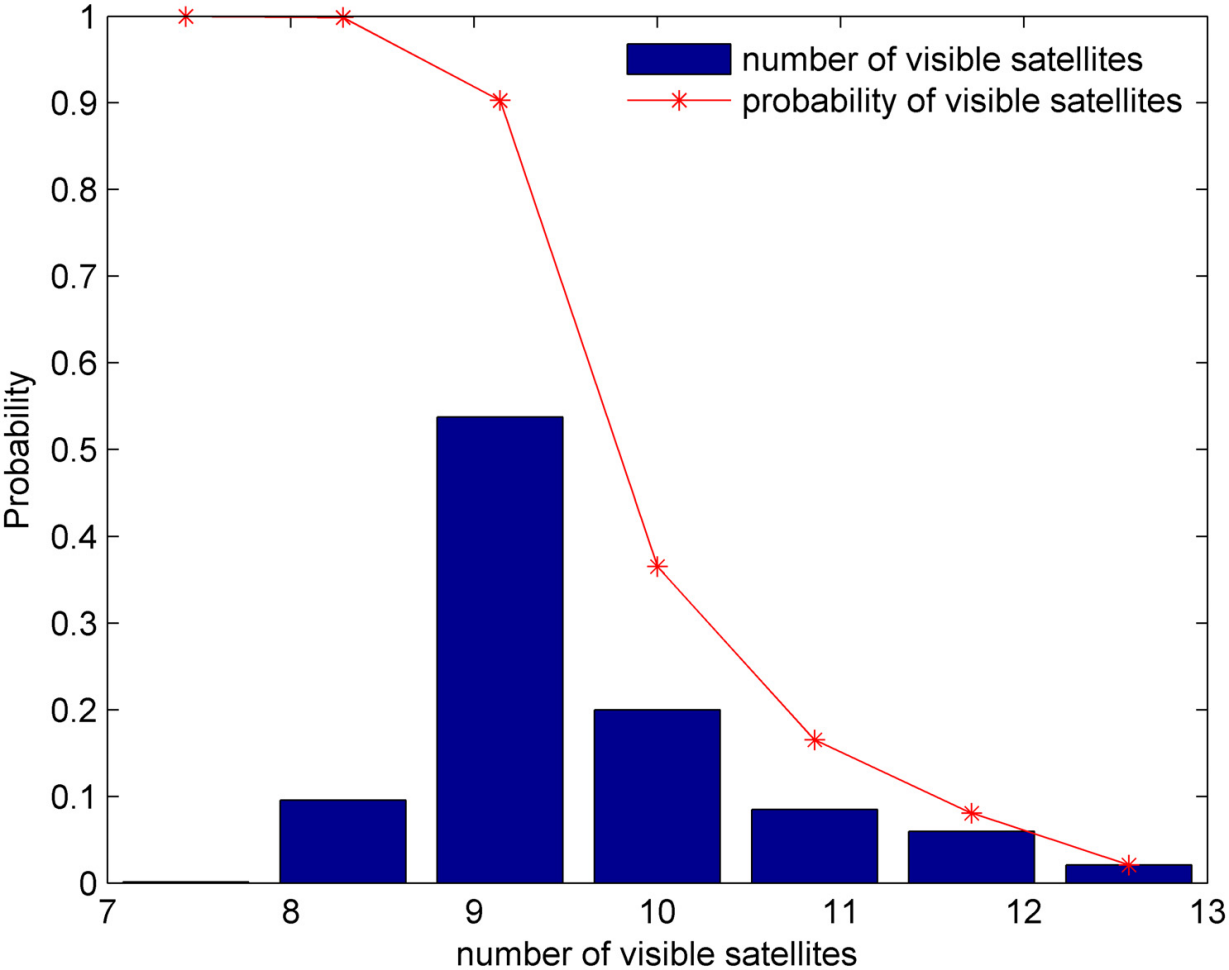
The 12-hour probability distribution at TianJin.

**Fig 5:**
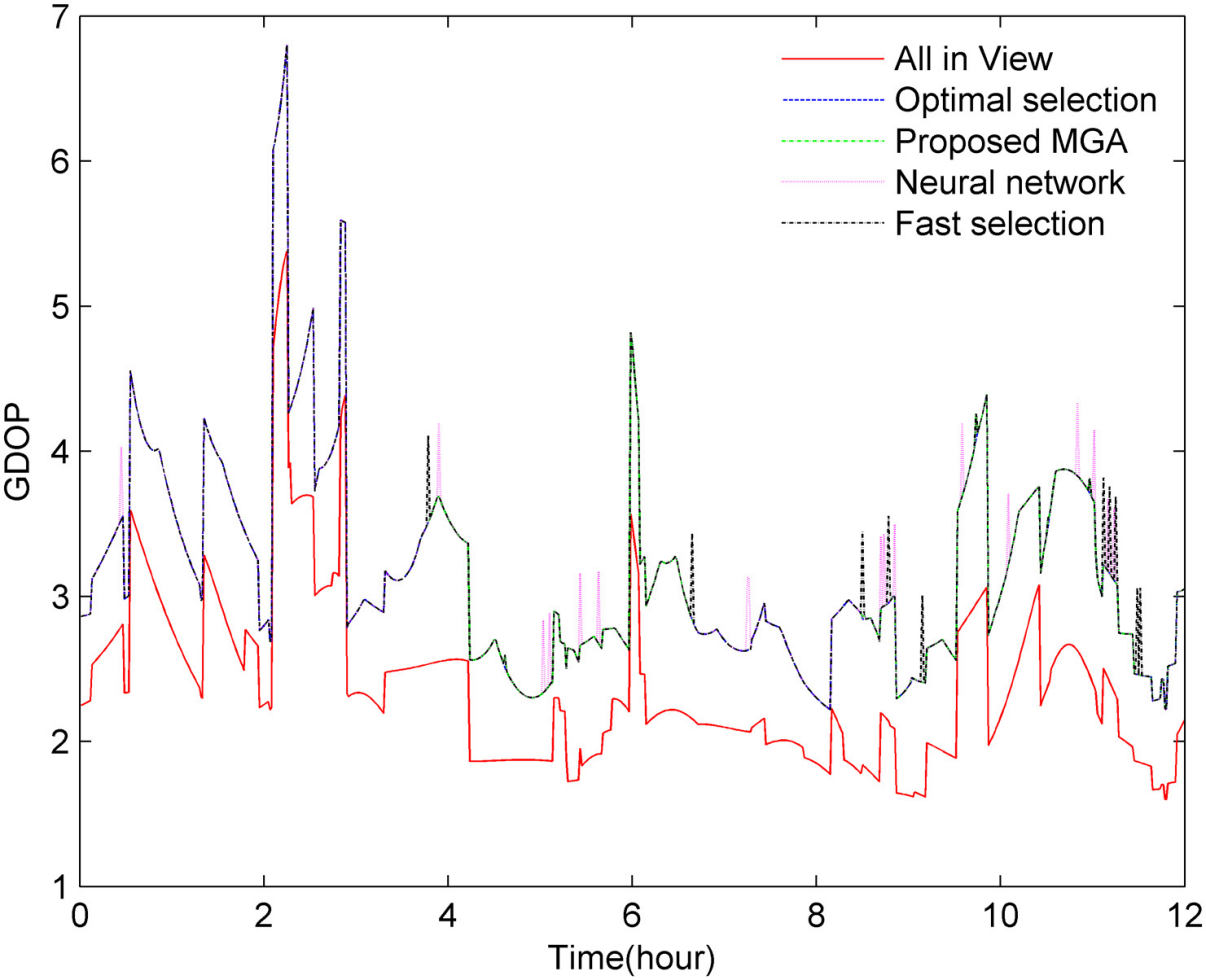
The performance of the MGA compared with that of using the AIV-method, a neural network, optimal selection and fast selection.

**Fig 6:**
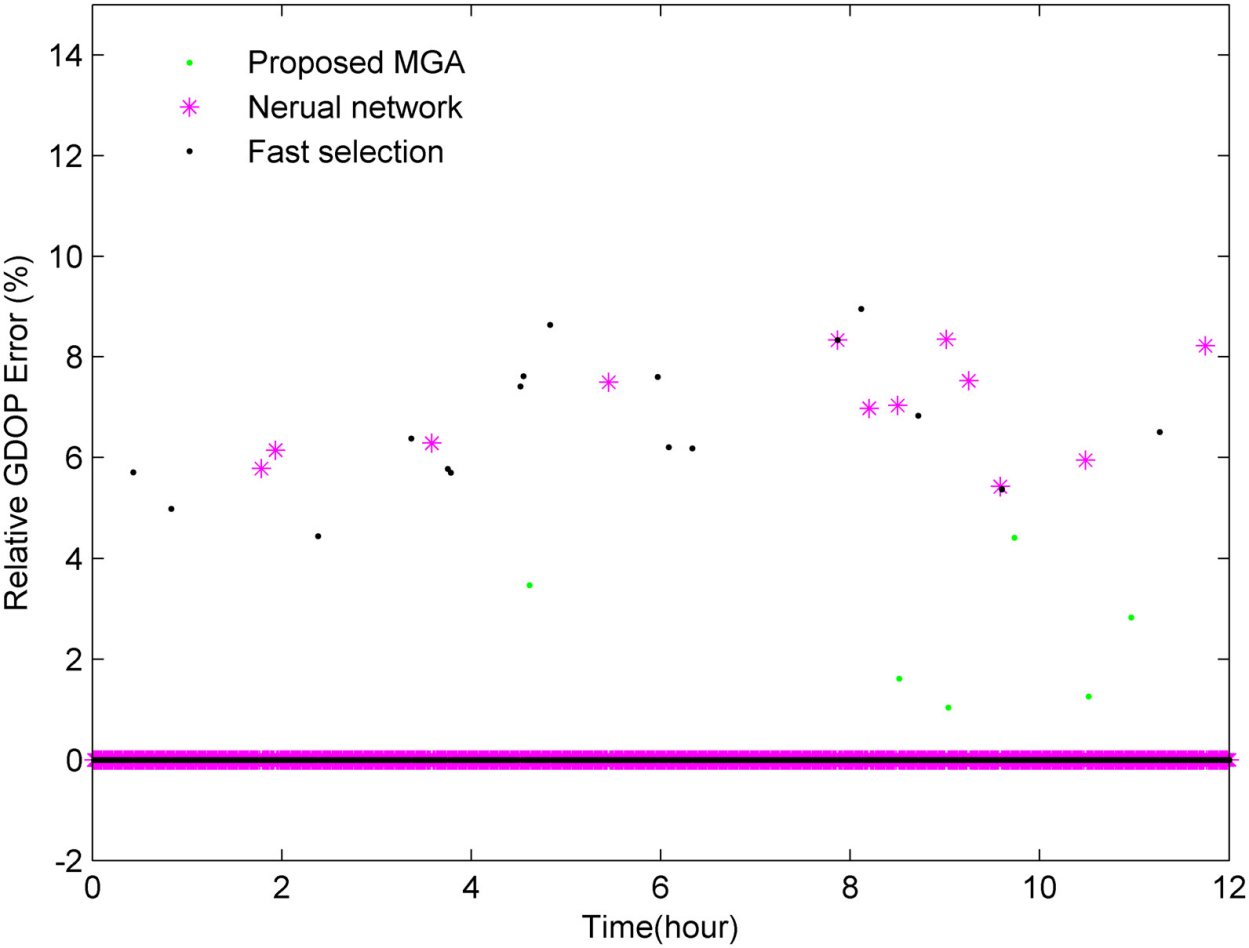
The relative error in the GDOP when the MGA is used for satellite selection.

**Fig 7:**
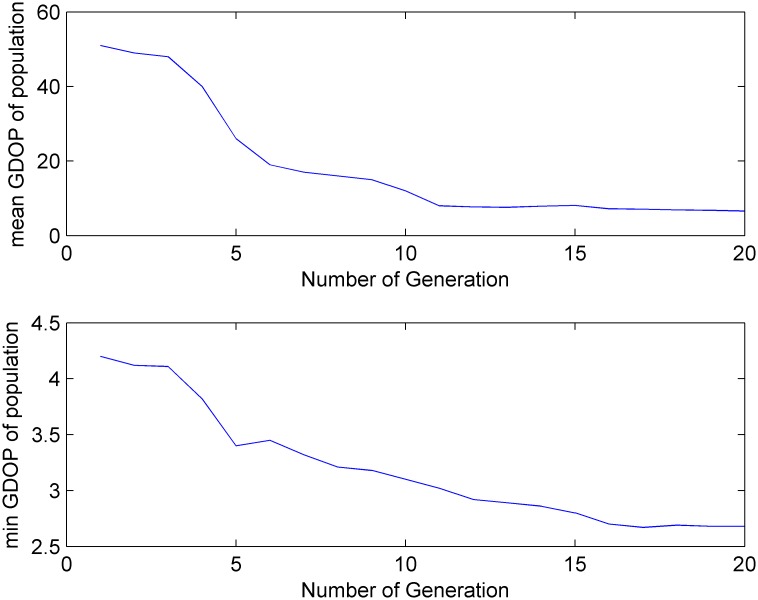
The maximum and minimum GDOP of each generation.

GPS satellites orbit the earth once approximately every 12 hours, and as shown in Fig 3, there are approximately 7–12 satellites visible from any point on the earth at any given time. Fig 4 shows the cumulative probability distribution function for the number of visible satellites. Fig 5 shows that the GDOP calculated using the all-in-view (AIV) method is minimized, and the proposed MGA’s performance is equivalent to that of the optimal selection algorithm. The GDOP of the optimal selection algorithm is slightly better than that of the AIV method, whose performance, however, is better than that of the neural network and the fast selection algorithm, particularly in terms of accuracy and efficiency. A comparison is shown in Table 2. A comparison of the residuals of the GDOPs of the various algorithms is provided in Fig 6, where it can be observed that the proposed MGA is the most accurate and has the smallest residuals. Fig 7 shows the average fitness function and the best individual fitness versus the number of iterations. The average fitness function gradually decreases over time as the process of evolution continues, which shows that the algorithm converges and that the best chromosome is eventually obtained.

**Table 2:** A comparison of the results.

Approach	Correction Percent(%)	Minimum GDOP	Maximum GDOP	Average GDOP	Time(s)
Optimal selection	100	2.12	6.79	3.16	8.38
Proposed MGA	99.6	2.12	6.82	3.19	0.146
Neural Network	92.9	2.38	7.14	3.34	0.168
Fast selection	93.2	2.32	7.23	3.32	0.25

**Fig 8:**
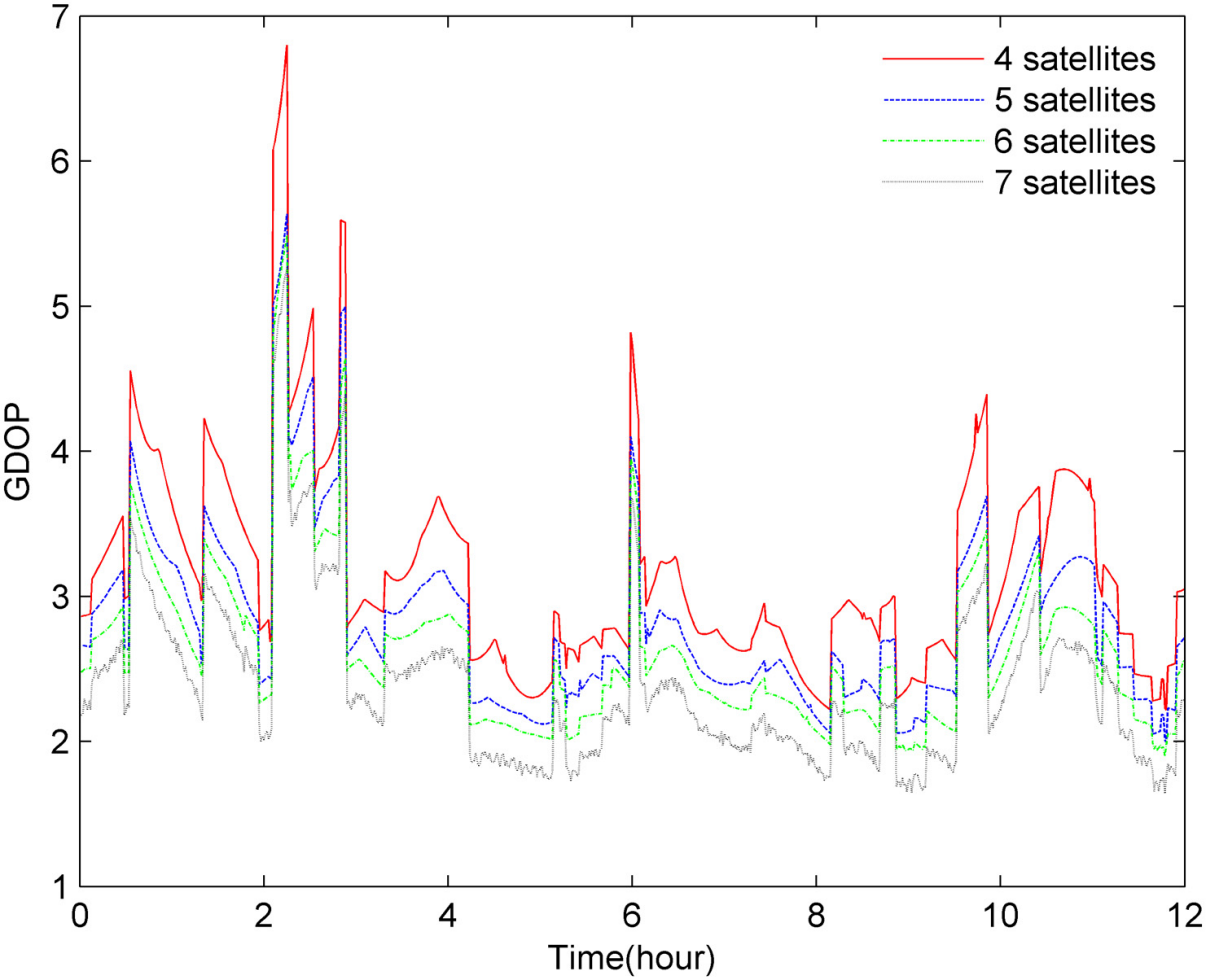
A comparison of the GDOP calculated using different numbers of satellites selected using the MGA.

The proposed novel MGA satellite selection algorithm is not only suitable for the common problem of selecting 4 satellites but can also be used to select 5 or 6 satellites under RAIM or FDE conditions without modification. Fig 8 presents the GDOP performance curve computed by the MGA, which shows that the GDOP gradually decreases as the number of satellites selected, namely, 4, 5 and 6, increases.

## Conclusion

A novel method for selecting satellites for GPS use was proposed and shown to provide both parallel and global convergence. The MGA can select a subset of satellites of any size that satisfies the position requirements using an elite conservation strategy, adaptive mechanism, hybrid genetic algorithm and reasonable designs of the fitness function, and the selection, crossover and mutation operators. Comprehensive simulations were conducted and demonstrated that the MGA-based satellite selection method can effectively select an optimal subset of the available satellites in both conventional and RAIM modes. The latter is more feasible and adaptable to the GPS receivers that are used in handset equipment and mobile phones. An unexpected discovery was that this method can be applied to not only single constellation systems such as GPS but also to multi-constellation systems because of its ability to select more than 4 satellites.

## Supporting Information

S1 Text This file contains the data (in the file format of matlab *.mat) collected by the authors at Tianjin, China for this study. (MAT)

S2 Text This file is description of used the data set (S1 Text) for satellite selection. (TXT)
